# Novel SARS-CoV-2 Variant Derived from Clade 19B, France

**DOI:** 10.3201/eid2705.210324

**Published:** 2021-05

**Authors:** Slim Fourati, Jean-Winoc Decousser, Souraya Khouider, Melissa N’Debi, Vanessa Demontant, Elisabeth Trawinski, Aurélie Gourgeon, Christine Gangloff, Grégory Destras, Antonin Bal, Laurence Josset, Alexandre Soulier, Yannick Costa, Guillaume Gricourt, Bruno Lina, Raphaël Lepeule, Jean-Michel Pawlotsky, Christophe Rodriguez

**Affiliations:** Institut Mondor de Recherche Biomédicale, Université Paris-Est, Créteil, France; (S. Fourati, S. Khouider, A. Gourgeon, A. Soulier, J.-M. Pawlotsky, C. Rodriguez);; Hôpital Henri Mondor, Créteil (J.-W. Decousser, M. N’Debi, V. Demontant, E. Trawinski, G. Gricourt, R. Lepeule, C. Rodriguez);; Hôpital Albert Chenevier, Créteil (C. Gangloff);; Université de Lyon, France (G. Destras, A. Bal, L. Josset, B. Lina);; Grand Hôpital de l’Est Francilien, Jossigny, France (Y. Costa)

**Keywords:** COVID-19, coronavirus disease, SARS-CoV-2, severe acute respiratory syndrome coronavirus 2, viruses, respiratory infections, zoonoses, HMN.19B variant, Henri Mondor variant, clade, France

## Abstract

We report a novel severe acute respiratory syndrome coronavirus 2 variant derived from clade 19B (HMN.19B variant or Henri Mondor variant). This variant is characterized by the presence of 18 amino acid substitutions, including 7–8 substitutions in the spike protein and 2 deletions. These variants actively circulate in different regions of France.

During fall 2020, new severe acute respiratory syndrome coronavirus 2 (SARS-CoV-2) variants, some of which have become variants of concern, progressively replaced the original strains in regions where they were first identified. We report a new SARS-CoV-2 variant of interest derived from clade 19B (tentatively named HMN.19B variant, or Henri Mondor variant) that is actively circulating in France.

On January 21, 2021, a hospital administrative assistant receiving long-term treatment with anti–tumor necrosis factor-α (adalimumab) for ankylosing spondylitis sought treatment for headache, fatigue, and rhinitis suggestive of coronavirus disease (COVID-19). SARS-CoV-2 RNA was confirmed by reverse transcription PCR (RT-PCR). Her partner (household contact), along with 2 nurses from the same occupational health unit sharing their locker room with the administrative assistant, sought treatment for symptoms suggestive of COVID-19 during January 21–23. Virus was confirmed in all instances by RT-PCR.

The slightly immunocompromised administrative assistant and her immunocompetent partner reported a history of symptomatic COVID-19 infection in early October 2020, confirmed in both cases by a positive RT-PCR result. However, both patients tested negative for SARS-CoV-2 protein N antibodies in January 2021. One of the 2 infected nurses had received a first dose of COVID-19 vaccine (Pfizer-BioNTech, https://www.pfizer.com) 11 days before her positive RT-PCR result. All 4 patients experienced mild COVID-19 and did not require hospitalization.

Full-length genome sequencing revealed that the 4 cluster members were infected with a new phylogenetic variant stemming from clade 19B, tentatively called HMN.19B variant or Henri Mondor variant (Appendix). Compared with the reference sequence (GenBank accession no. NC_045512.2) from the international GISAID database (https://www.gisaid.org), variant HMN.19B carries 25 nt substitutions, with a high ratio of nonsynonymous (n = 18) to synonymous (n = 7) mutations, 2 deletions, and a high number of amino acid substitutions within the spike protein (n = 8) at key positions: spike substitutions in comparison with other recently emerged variants (Table) and all mutations (Figure).

**Table:** Amino acid changes within the spike protein sequence of the HMN.19B variant in France compared with the reference sequence of the international GISAID database (GenBank accession no. NC_045512.2), in comparison with other recently identified SARS-CoV-2 variants*

Spike protein sequence				
HMN.19B (*Henri Mondor variant)*	20I/501Y.V1 (UK variant)	20H/501Y.V2 (South African variant)	P1 20J/501Y.V3 (Brazilian variant)	CAL.20C (Californian variant)
				S13I
**L18F**		L18F	L18F	
			T20N	
			P26S	
	Del69/70			
		D80A		
			D138Y	
	Del144			
				W152C
			R190S	
		D215G		
		Del 242–244		
		R246I		
		K417N	K417T	
**L452R**				L452R
		E484K	E484K	
**N501Y**	N501Y	N501Y	N501Y	
	A570D			
A653V				
**H655Y**			H655Y	
Q677H†				
	P681H			
		A701V		
	T716I			
D796Y				
	S982A			
			T1027I	
	D1118H			
G1219V				

*Bold type indicates amino acid changes observed in *>*1 of the recent variants. *†Inconstantly detected, recently found in the genome of the “Midwest” variant (Q677H variant) observed in Ohio (USA) in December 2020 and January 2021.*

**Figure:**
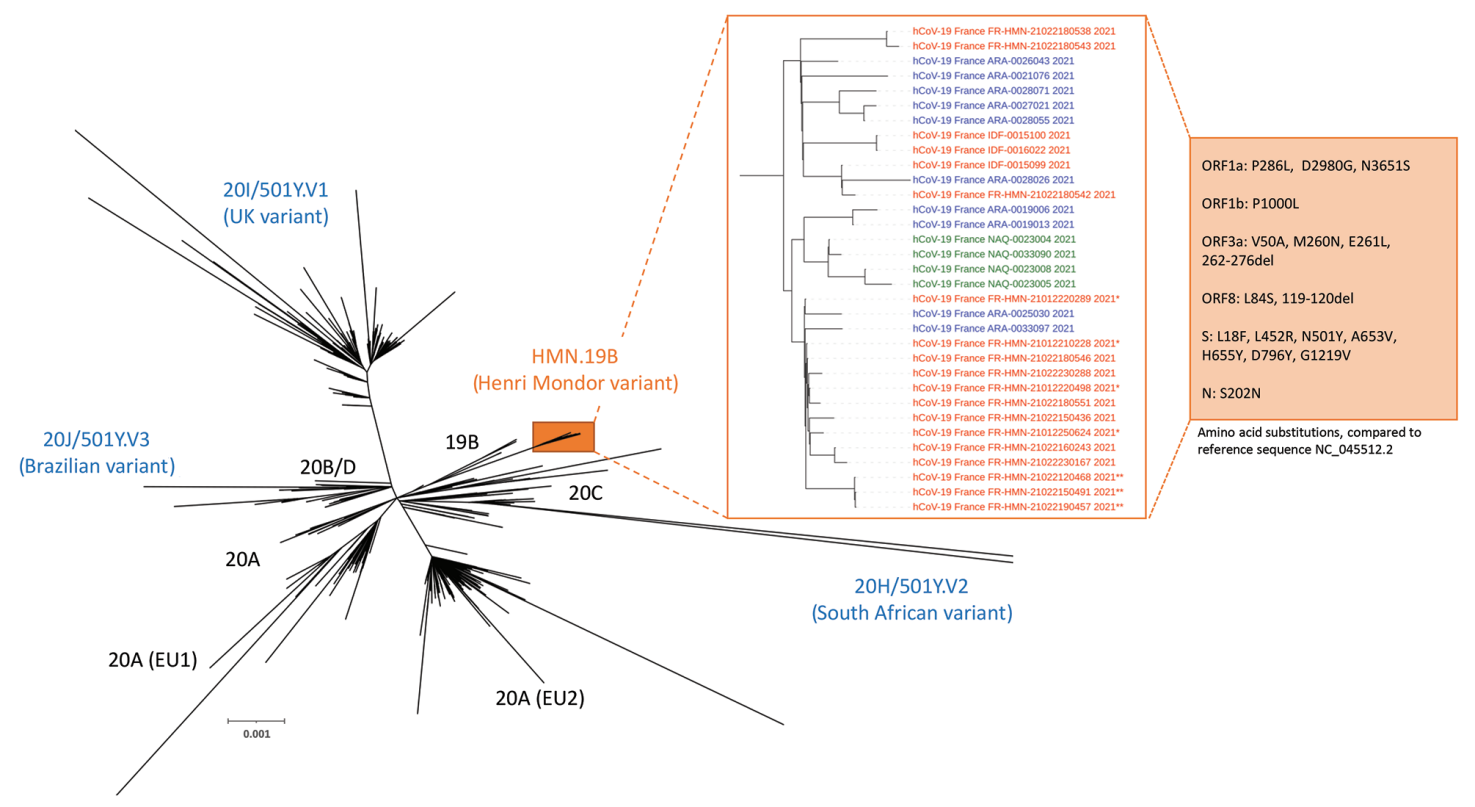
Phylogenetic tree built with sequences from the 33 patients infected with the new HMN.19B or Henri Mondor variant and from 1,537 SARS-CoV-2–infected patients in France sampled during January 18–February 23, 2021, sequenced in 9 successive series. Phylogeny was performed after full-length genome alignment with Muscle 3.8.31 (maximum-likelihood model general time-reversible plus invariant sites model, 1,000 bootstrap replicates) by means of IQ-Tree 1.3.11.1 and iTOL. The HMN.19B (Henri Mondor) variant cluster is considerably different from all the others, with a 99% bootstrap value. HMN.19B sequences are colored according to the geographic origin of the patients: red, greater Paris area (IDF or HMN); blue, southeast France (ARA); green, southwest France (NAQ). *Original cluster in an occupational health unit; **cluster in the hematology department of our institution. Amino-acid substitutions and deletions detected in all sequences are described in the orange box. ARA, Auvergne-Rhône-Alpes; HMN, Henri Mondor; IDF, Ile-de-France; NAQ, Nouvelle-Aquitaine.

In the 4 weeks after its first detection, our laboratory, which maintains 1 of the 4 national SARS CoV-2 sequencing surveillance platforms in France, found the HMN.19B variant in 12 patients from the greater Paris area (Figure). These patients were 1 prison administration staff member from northeast of the Paris area, tested February 9 during a prison screening campaign; 3 epidemiologically related subjects from a cluster in the hematology department of our hospital (an asymptomatic nursing student tested February 12, his mentor nurse tested February 14, and a hospitalized patient tested February 15); and 8 epidemiologically unrelated cases found positive for SARS-CoV-2 RNA during February 3–23 in different hospitals in the greater Paris area (GISAID identification numbers in Appendix Table).

During the same period, the National Reference Center for Respiratory Viral Infections (Lyon, France) identified 17 additional patients infected with closely related viruses, which carried >7 similar substitutions in spike (some were lacking Q677H in spike [Figure]). Three patients were from the greater Paris area, 10 from southeastern France, and 4 from southwestern France (Figure).

We identified a new, previously undescribed variant of SARS-CoV-2 (HMN.19B or Henri Mondor variant) within a cluster of hospital staff in Paris. This variant stems from an older SARS CoV-2 clade, 19B, which emerged in late 2019 but have been rarely detected since early 2020, overtaken by clades 20A, 20B, and 20C, which harbor the D614G substitution believed to improve viral transmission (*1*). The HMN.19B variant is characterized by the presence of 2 deletions and 18 amino acid substitutions over the entire sequence, including 8 substitutions within the spike protein, some of which are common with other recently described variants, a finding in keeping with the ongoing evolutionary convergence of SARS-CoV-2 variants. The acquisition of spike substitutions, including N501Y and L452R, has been suggested to enhance the interaction of spike with the angiotensin-converting enzyme 2 viral receptor. The resulting substantial fitness acquisition could explain the reappearance of clade 19B (*2*; Yang et al., unpub. data, https://doi.org/10.1101/2020.12.29.42469). 

New variants with several spike mutations (20I/501Y.V1) have been associated with increased transmissibility. Whether HMN.19B will be less susceptible to protection by natural, therapeutic, or vaccine-induced immune responses remains to be determined. Several of its spike substitutions (N501Y, L452R, and H655Y) have been shown to require higher levels of neutralizing antibodies to be controlled, both in vitro and in vivo (*3**,**4*; Liu et al., unpub. data, https://doi.org/10.1101/2020.11.06.372037).

In conclusion, we report a new SARS-CoV-2 variant circulating in France. Our results emphasize the need for careful molecular surveillance of SARS-CoV-2 evolution to track emergence of any new variant of interest with potential epidemiologic or pathophysiologic consequences.

Appendix Additional information about novel SARS-CoV-2 variant derived from clade 19B, France.
