# Supplementary material for: Novel SARS-CoV-2 Variant Derived from Clade 19B, France
Source: Emerg Infect Dis. 2021 May;27(5):1540–3. doi: 10.3201/eid2705.210324 (PMC8084519; doi:10.3201/eid2705.210324)
Supplement: Appendix — Additional information about novel SARS-CoV-2 variant derived from clade 19B, France. [file 21-0324-Techapp-s1.pdf]

Article DOI: https://doi.org/10.3201/eid2705.210324

Novel SARS-CoV-2 Variant Derived from Clade 19B, France

Appendix

Methods

The full-length SARS-CoV-2 genomes from the 4 infected patients were sequenced by next-generation sequencing. Viral RNA was extracted from nasopharyngeal swabs with a NucliSENS easyMAG kit on an EMAG device (bioMérieux, https://www.biomerieux.com). Sequencing was performed with the COVIDSeq Test (Illumina, https://www.illumina.com), which uses 98-target multiplex amplifications along the full SARS-CoV-2 genome (Bhoyar et al. unpub. data, https://doi.org/10.1101/2020.08.10.242677). The libraries were sequenced with NextSeq 500/550 High Output Kit 2.5 on a NextSeq 500 device (Illumina). The sequences were demultiplexed and assembled as full-length genomes with the DRAGEN COVIDSeq Test Pipeline on a DRAGEN server (Illumina). Lineages and clades were interpreted with Pangolin and NextClade (*1*). Phylogeny was performed after full-length genome alignment with Muscle 3.8.31 (maximum-likelihood model general time-reversible plus invariant sites; 1,000 bootstrap replicates) by using IQ-Tree version 1.3.11.1 and iTOL (*2*,*3*).

References

1. Rambaut A, Holmes EC, O'Toole Á, Hill V, McCrone JT, Ruis C, et al. A dynamic nomenclature proposal for SARS-CoV-2 lineages to assist genomic epidemiology. Nat Microbiol. 2020;5:1403–7. PubMed https://doi.org/10.1038/s41564-020-0770-5

2. Minh BQ, Schmidt HA, Chernomor O, Schrempf D, Woodhams MD, von Haeseler A, et al. IQ-TREE 2: new models and efficient methods for phylogenetic inference in the genomic era. Mol Biol Evol. 2020;37:1530–4. PubMed https://doi.org/10.1093/molbev/msaa015

3. Letunic I, Bork P. Interactive Tree Of Life (iTOL) v4: recent updates and new developments. Nucleic Acids Res. 2019;47(W1):W256–9. PubMed https://doi.org/10.1093/nar/gkz239

Publisher: CDC; Journal: Emerging Infectious Diseases
Article Type: Research Letter; Volume: 27; Issue: 5; Year: 2021; Article ID: 21-0324
DOI: 10.3201/eid2705.210324; TOC Head: Research Letter

Appendix Table. Amino acid substitutions observed in the spike protein of the 19B.HMN variants*

	Amino acid position in spike protein													
Lab identification number submitted to GISAID	18	49	70	346	452	501	653	655	677	796	798	1113	1191	1219
hCoV-19/France/FR-HMN-21022180538/2021	L18F				L452R	N501Y	A653V	H655Y		D796Y	G798D			G1219V
hCoV-19/France/FR-HMN-21022180543/2021	L18F				L452R	N501Y	A653V	H655Y		D796Y	G798D			G1219V
hCoV-19/France/ARA-0026043/2021	L18F				L452R	N501Y	A653V	H655Y		D796Y				G1219V
hCoV-19/France/ARA-0021076/2021	L18F				L452R	N501Y	A653V	H655Y		D796Y				G1219V
hCoV-19/France/ARA-0028071/2021	L18F				L452R	N501Y	A653V	H655Y		D796Y				G1219V
hCoV-19/France/ARA-0027021/2021	L18F	H49Y			L452R	N501Y	A653V	H655Y		D796Y				G1219V
hCoV-19/France/ARA-0028055/2021	L18F	H49Y			L452R	N501Y	A653V	H655Y		D796Y				G1219V
hCoV-19/France/IDF-0015100/2021	L18F				L452R	N501Y	A653V	H655Y		D796Y				G1219V
hCoV-19/France/IDF-0016022/2021	L18F				L452R	N501Y	A653V	H655Y		D796Y				G1219V
hCoV-19/France/IDF-0015099/2021	L18F				L452R	N501Y	A653V	H655Y		D796Y				G1219V
hCoV-19/France/ARA-0028026/2021	L18F				L452R	N501Y	A653V	H655Y		D796Y				G1219V
hCoV-19/France/FR-HMN-21022180542/2021	L18F				L452R	N501Y	A653V	H655Y		D796Y				G1219V
hCoV-19/France/ARA-0019006/2021	L18F			R346I	L452R	N501Y	A653V	H655Y	Q677H	D796Y			K1191N	G1219V
hCoV-19/France/ARA-0019013/2021	L18F			R346I	L452R	N501Y	A653V	H655Y	Q677H	D796Y			K1191N	G1219V
hCoV-19/France/NAQ-0023004/2021	L18F				L452R	N501Y	A653V	H655Y	Q677H	D796Y			K1191N	G1219V
hCoV-19/France/NAQ-0033090/2021	L18F		V70F		L452R	N501Y	A653V	H655Y	Q677H	D796Y			K1191N	G1219V
hCoV-19/France/NAQ-0023008/2021	L18F				L452R	N501Y	A653V	H655Y	Q677H	D796Y			K1191N	G1219V
hCoV-19/France/NAQ-0023005/2021					L452R	N501Y	A653V	H655Y	Q677H	D796Y			K1191N	G1219V
hCoV-19/France/FR-HMN-21012220289/2021	L18F				L452R	N501Y	A653V	H655Y	Q677H	D796Y				G1219V
hCoV-19/France/ARA-0025030/2021	L18F				L452R	N501Y	A653V	H655Y	Q677H	D796Y				G1219V
hCoV-19/France/ARA-0033097/2021	L18F				L452R	N501Y	A653V	H655Y	Q677H	D796Y		Q1113K		G1219V
hCoV-19/France/FR-HMN-21012210228/2021	L18F				L452R	N501Y	A653V	H655Y	Q677H	D796Y				G1219V
hCoV-19/France/FR-HMN-21022180546/2021	L18F				L452R	N501Y	A653V	H655Y	Q677H	D796Y				G1219V
hCoV-19/France/FR-HMN-21022230288/2021					L452R	N501Y	A653V	H655Y	Q677H	D796Y				G1219V
hCoV-19/France/FR-HMN-21012220498/2021	L18F				L452R	N501Y	A653V	H655Y	Q677H	D796Y				G1219V
hCoV-19/France/FR-HMN-21022180551/2021	L18F				L452R	N501Y	A653V	H655Y	Q677H	D796Y				G1219V
hCoV-19/France/FR-HMN-21022150436/2021	L18F				L452R	N501Y	A653V	H655Y	Q677H	D796Y				G1219V
hCoV-19/France/FR-HMN-21012250624/2021	L18F				L452R	N501Y	A653V	H655Y	Q677H	D796Y				G1219V
hCoV-19/France/FR-HMN-21022160243/2021	L18F				L452R	N501Y	A653V	H655Y	Q677H	D796Y				G1219V
hCoV-19/France/FR-HMN-21022230167/2021	L18F				L452R	N501Y	A653V	H655Y	Q677H	D796Y				G1219V
hCoV-19/France/FR-HMN-21022120468/2021	L18F				L452R	N501Y	A653V	H655Y	Q677H	D796Y				G1219V
hCoV-19/France/FR-HMN-21022150491/2021	L18F				L452R	N501Y	A653V	H655Y	Q677H	D796Y				G1219V
hCoV-19/France/FR-HMN-21022190457/2021	L18F				L452R	N501Y	A653V	H655Y	Q677H	D796Y				G1219V

*HMN.19B spike sequences are colored based on geographic region of origin of patients: greater Paris area (IDF or HMN) in red, southeast France (ARA) in blue, and southwest France (NAQ) in green.
